# Novel HDAC inhibitor MAKV-8 and imatinib synergistically kill chronic myeloid leukemia cells via inhibition of BCR-ABL/MYC-signaling: effect on imatinib resistance and stem cells

**DOI:** 10.1186/s13148-020-00839-z

**Published:** 2020-05-19

**Authors:** Manon Lernoux, Michael Schnekenburger, Hélène Losson, Koen Vermeulen, Hyunggu Hahn, Déborah Gérard, Jin-Young Lee, Aloran Mazumder, Muneer Ahamed, Christo Christov, Dong-Wook Kim, Mario Dicato, Guy Bormans, Byung Woo Han, Marc Diederich

**Affiliations:** 1 Laboratoire de Biologie Moléculaire et Cellulaire du Cancer, Hôpital Kirchberg, 9, rue Edward Steichen, L-2540 Luxembourg, Luxembourg; 2 Laboratory for Radiopharmaceutical Research, Department of Pharmaceutical and Pharmacological Sciences, KU Leuven, Leuven, Belgium; 3 Department of Pharmacy, College of Pharmacy, Seoul National University, 1 Gwanak-ro, Gwanak-gu, Seoul, 08826 Korea; 4 Faculté de Médecine, Université de Lorraine, Nancy, France; 5 Seoul St. Mary’s Hospital, Leukemia Research Institute, the Catholic University of Korea, Seoul, Korea

**Keywords:** Epigenetic regulation, Tyrosine kinase inhibitor, Computational docking, Autophagy, Apoptosis, Endoplasmic reticulum stress

## Abstract

**Background:**

Chronic myeloid leukemia (CML) pathogenesis is mainly driven by the oncogenic breakpoint cluster region-Abelson murine leukemia viral oncogene homolog 1 (BCR-ABL) fusion protein. Since BCR-ABL displays abnormal constitutive tyrosine kinase activity, therapies using tyrosine kinase inhibitors (TKis) such as imatinib represent a major breakthrough for the outcome of CML patients. Nevertheless, the development of TKi resistance and the persistence of leukemia stem cells (LSCs) remain barriers to cure the disease, justifying the development of novel therapeutic approaches. Since the activity of histone deacetylase (HDAC) is deregulated in numerous cancers including CML, pan-HDAC inhibitors may represent promising therapeutic regimens for the treatment of CML cells in combination with TKi.

**Results:**

We assessed the anti-leukemic activity of a novel hydroxamate-based pan-HDAC inhibitor MAKV-8, which complied with the Lipinski’s “rule of five,” in various CML cells alone or in combination with imatinib. We validated the in vitro HDAC-inhibitory potential of MAKV-8 and demonstrated efficient binding to the ligand-binding pocket of HDAC isoenzymes. In cellulo, MAKV-8 significantly induced target protein acetylation, displayed cytostatic and cytotoxic properties, and triggered concomitant ER stress/protective autophagy leading to canonical caspase-dependent apoptosis. Considering the specific upregulation of selected HDACs in LSCs from CML patients, we investigated the differential toxicity of a co-treatment with MAKV-8 and imatinib in CML versus healthy cells. We also showed that beclin-1 knockdown prevented MAKV-8-imatinib combination-induced apoptosis. Moreover, MAKV-8 and imatinib co-treatment synergistically reduced BCR-ABL-related signaling pathways involved in CML cell growth and survival. Since our results showed that LSCs from CML patients overexpressed c-MYC, importantly MAKV-8-imatinib co-treatment reduced c-MYC levels and the LSC population. In vivo, tumor growth of xenografted K-562 cells in zebrafish was completely abrogated upon combined treatment with MAKV-8 and imatinib.

**Conclusions:**

Collectively, the present findings show that combinations HDAC inhibitor-imatinib are likely to overcome drug resistance in CML pathology.

## Background

Chronic myeloid leukemia (CML) is a clonal myeloproliferative malignancy accounting for 15% of newly diagnosed leukemia cases in adults [1]. CML pathogenesis is mainly driven by the translocation t(9;22)(q34;11) between the breakpoint cluster region (BCR) and the Abelson murine leukemia viral oncogene homolog 1 (ABL) genes. The resulting fusion gene is translated into the oncogenic BCR-ABL protein with abnormal constitutive tyrosine kinase activity, which stimulates tumor cell proliferation and survival [2]. Accordingly, BCR-ABL-positive CML patients are currently treated with tyrosine kinase inhibitors (TKis) including imatinib. TKis effectively block downstream BCR-ABL signaling pathways and eliminate most CML cells [3]. Nevertheless, such therapeutic regimens are associated with severe side effects, as well as the development of TKi resistance, partly due to the reservoir of TKi-insensitive quiescent leukemic stem cells (LSCs) [4, 5]. Therefore, novel therapeutic approaches are required to overcome these limitations and effectively cure CML patients. Combining imatinib with epigenetic modulators has emerged as a promising strategy for improving anti-leukemic therapy. Accordingly, TKi-HDACi combinations have been shown to induce synergistic anti-CML effects and LSC eradication [6].

Histone deacetylases (HDACs) catalyze the removal of acetyl groups from lysine residues of various histones and non-histone protein targets. They thus act as important regulators of gene expression and are implicated in a plethora of cellular processes [7]. Nowadays, it is established that aberrant activation or overexpression of HDAC isoenzymes trigger disruptions of the functional acetylation landscape, therefore contributing to the development of numerous cancers including CML [8, 9]. Since HDACs are considered to be attractive targets for cancer prevention and therapy, pan-HDAC inhibitors (HDACis) represent a powerful class of epigenetically active therapeutic drugs that have already demonstrated promising anti-cancer activities in pre-clinical studies and are undergoing clinical trials for many cancers [10, 11]. Four HDACis have achieved Food and Drug Administration approval: class I selective- (FK-228) or pan-HDACis [suberoylanilide hydroxamic acid (SAHA), PXD-101, and LBH-589] [12]. In particular, SAHA has been repeatedly reported to enhance the cytotoxicity of various chemotherapeutic drugs including TKis such as imatinib [13] and dasatinib [14].

MAKV-8, characterized by a linker of 6-methylene units and a CAP group with arylisoxazole, has been initially reported to display an IC_50_ value of 2 pM towards HDAC3 and HDAC6 in vitro, and its anti-proliferative activity against pancreatic cancer cells was similar to that of SAHA [15]. However, its HDAC-inhibitory properties were never tested in cellulo, and the molecular mechanism through which MAKV-8 exhibits anti-cancer effects has not been characterized. In this study, we demonstrated that the pan-HDACi MAKV-8 in combination with imatinib displays anti-leukemic properties, which are likely to lower the burden of resistance in CML pathology.

## Results

### In vitro HDAC inhibition by MAKV-8

**Fig. 1:**
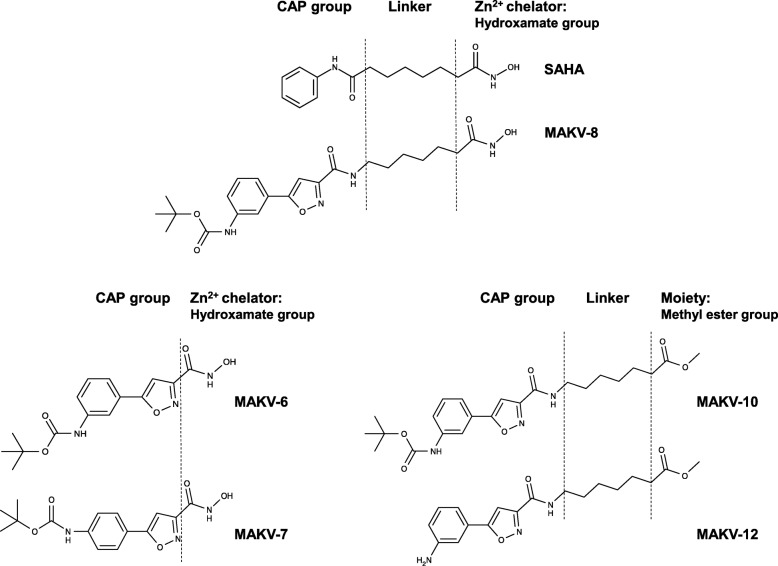
Chemical structures of MAKV-6, MAKV-7, MAKV-8, MAKV-10, and MAKV-12 and the reference HDACi, SAHA. The prototypical pharmacophoric model of an HDACi is constituted by the zinc binding group, the hydrophobic linker region, and the cap group. MAKV-6 and MAKV-7 lack the linker portion; MAKV-10 and MAKV-12 substitute the hydroxamate group with a methyl ester group and were obtained as synthesis intermediates

**Fig. 2:**
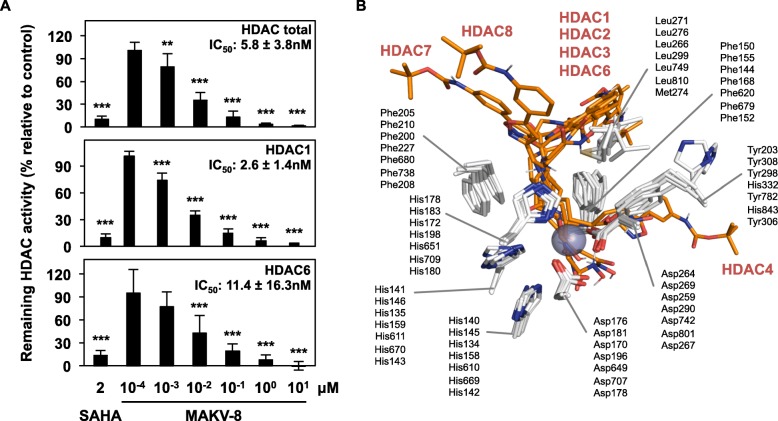
MAKV-8 inhibits HDAC activities in vitro and binds to the ligand-binding pocket of HDAC isoenzymes. (**a**) In vitro HDAC activity assays were conducted with increasing MAKV-8 concentrations. Relative activities of total HDAC, HDAC1, and HDAC6 were determined by comparison to the vehicle, DMSO. (**b**) Docking poses of MAKV-8 (stick model, orange) on the crystal structure of indicated HDAC isoenzymes (white; PDB codes: see Methods section). Numbered residues forming hydrophobic interactions in the binding sites (stick representation) correspond to HDAC1 to HDAC8 from top to bottom. Zinc atom is shown as a purple sphere; nitrogen and oxygen are colored in blue and red, respectively

First, we assessed the in vitro HDAC inhibitory potential of MAKV-8 using SAHA as a reference compound (Fig. 1). MAKV-8 inhibited total HDAC, as well as recombinant HDAC1 and HDAC6 deacetylase activities with IC_50_ values of 5.8, 2.6, and 11.4 nM, respectively, suggesting the inhibition of multiple deacetylase activities. Notably, about 10% of the total HDAC activity remained with 2 μM SAHA or 0.1 μM MAKV-8 (Fig. 2a).

### In silico prediction of the drug-likeness characteristics of MAKV-8

In silico predictions showed that MAKV-8 had low lipophilicity, as characterized by a miLog*P* coefficient below 5 and a logD_7.4_ of 2.8, which is a major criterion for orally active drugs. This compound expressed a topological polar surface area of 142.79 combined with a molecular weight of 446.5 Da; further, 4 and 10 hydrogen bond donors and acceptors, respectively, were recognized. These parameters imply free diffusion over the cell membrane. Interestingly, MAKV-8 displayed a favorable intestinal absorption parameter and plasma protein binding potential compared to PXD-101, predicting a good bioavailability (Table 1). Altogether, MAKV-8 displayed favorable drug-likeness parameters and a low predicted toxicity risk, similar to FDA-approved pan-HDACis.

**Table 1:** In silico predictions of MAKV-8 drug-likeness and oral bioavailability

Method	Parameter (unit)	Values				
		Theoretical	MAKV-8	SAHA	PXD-101	LBH-589
Lipinski’s rule of five	Volume (Å^3^)	NA	411.02	255.64	266.11	330.62
	miLog*P*	≤ 5	3.49	2.47	2.19	3.19
	MW (Da)	≤ 500	446.5	264.32	318.35	349.43
	n-OHNH	≤ 5	4	3	3	4
	n-ON	≤ 10	10	5	6	5
Ghose filter	n-atoms	20 ≤ × ≤ 70	32	19	22	26
Veber’s rule	n-rotb	≤ 10	12	8	5	7
	TPSA (Å^2^)	≤ 140	142.79	78.42	95.5	77.14
Absorption	BBBP	0.1 ≤ MA ≤ 2	0.12	0.22	0.18	1.16
	IA (%)	≥ 70	76.68	84.53	89.94	89.23
	PPB (%)	< 90	82.82	72.16	94.26	78.3
Toxicity	Rat	NA	Negative	Negative	Negative	Negative

*BBBP* blood-brain barrier penetration, *IA* intestinal absorption, *MA* middle absorption, *miLogP* octanol-water partition coefficient, *MW* molecular weight, *n-atoms* number of atoms, *n-OHNH* number of hydrogen bond donors, *n-ON* number of hydrogen bond acceptors, *n-rotb* number of rotatable bonds, *NA* not applicable, *PPB* plasma protein binding, *TPSA* topological polar surface area

### MAKV-8 efficiently binds to the ligand-binding pocket of HDAC isoenzymes

A docking simulation on a panel of human HDAC isoforms frequently associated with tumorigenesis indicated that the hydroxamate group and hydrophobic linker region of MAKV-8 established efficient interactions in the ligand-binding pocket of all HDAC isoenzymes, whereas its CAP group interacted with loops around the ligand-binding pocket (Fig. 2b; Additional file 1: Figure S1). Qualitative molecular analyses demonstrated that MAKV-8 displayed more potent binding affinities than SAHA for all tested HDACs, with average values of − 7.1 and − 6.2 kcal/mol, respectively, and suggested a moderately different HDAC-inhibitory profile between MAKV-8 and SAHA, since binding affinity energy values were similar for certain HDACs and distinct for others (Table 2).

**Table 2:** Qualitative molecular docking of MAKV-8 against selected HDACs

HDAC (PDB code)	MAKV-8	SAHA
**HDAC1 (4BKX)**	− 6.7	− 5.4
**HDAC2 (4LY1)**	− 7.2	− 6.7
**HDAC3 (4A69)**	− 6.9	− 6.5
**HDAC4 (2VQM)**	− 7.7	− 5.6
**HDAC6 (5EDU)**	− 7.2	− 6.1
**HDAC7 (3C10)**	− 7.1	− 6.0
**HDAC8 (3EW8)**	− 7.0	− 6.9
**Average**	− 7.1	− 6.2

Binding affinity energy values (kcal/mol) for the indicated Protein Data Bank (PDB) codes were calculated using AutoDock Vina program. SAHA was used as a reference HDACi

### MAKV-8 significantly induced target protein acetylation

**Fig. 3:**
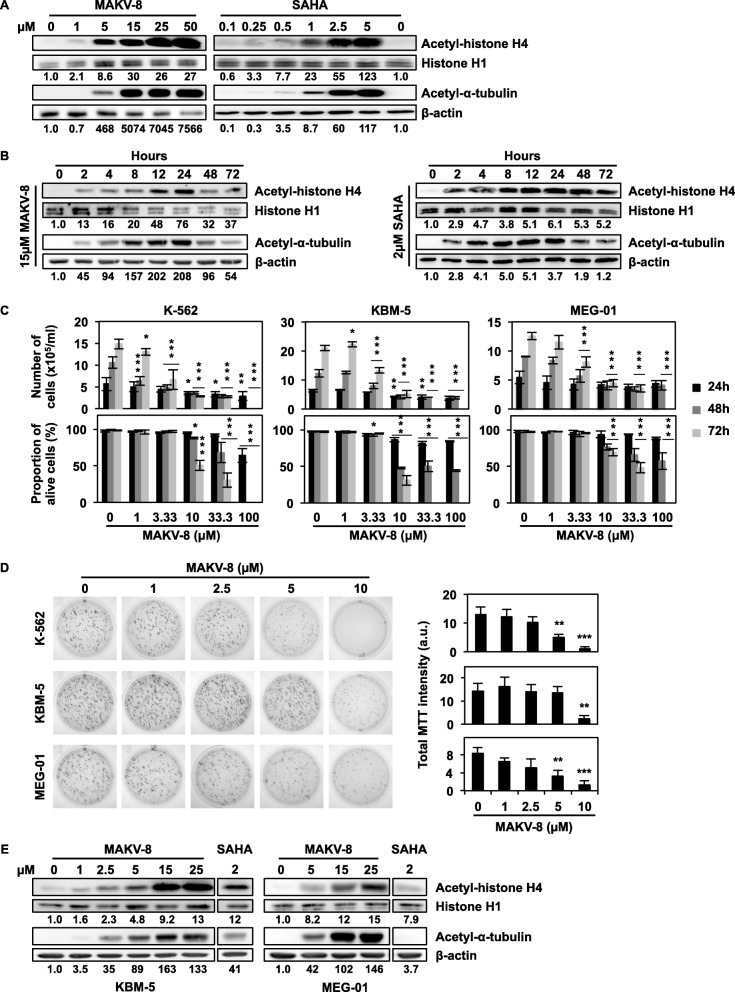
The potent pan-HDAC inhibitor MAKV-8 displays cytotoxic properties in CML cells. The acetylation levels of HDAC targets were assessed by western blot in K-562 cells treated with (**a**) increasing MAKV-8 concentrations for 24h or (**b**) 15µM MAKV-8 for the indicated time points. (**c**) CML cell proliferation and viability were evaluated following treatments with increasing MAKV-8 concentrations for up to 72h. (**d**) CML cells were grown in the presence of increasing MAKV-8 concentrations for 10 days, and their colony-forming capacity was scored after MTT addition. Representative pictures (left panel) and corresponding quantifications (right panel) from three independent experiments are provided. (**e**) Histone H4 and α-tubulin acetylation levels were assessed by western blot in KBM-5 and MEG-01 cells treated with increasing MAKV-8 concentrations for 24h. β-actin and histone H1 served as loading controls for α-tubulin and histone H4, respectively. Blots are representative of three independent experiments. SAHA was used as a reference HDACi

To determine whether MAKV-8 acted as an HDACi in cellulo, we next analyzed its effect compared to SAHA on the acetylation of histone H4, a nuclear substrate of class I, II, and IV HDACs, and α-tubulin, a cytoplasmic substrate of HDAC6. In K-562 cells, MAKV-8 strongly induced α-tubulin and histone H4 acetylation in a concentration-dependent manner, starting from 5 and 1 μM, respectively (Fig. 3a). SAHA increased such protein acetylation in a similar manner to that observed with MAKV-8, albeit at lower concentrations. Noteworthy, EC_50_ values suggested that MAKV-8 displayed increased selectivity against nuclear HDACs targeting histone H4 compared to HDAC6 targeting α-tubulin, whereas SAHA acted similarly on both targets (Table 3). Next, kinetic analysis of α-tubulin and histone H4 acetylation status showed a rapid and time-dependent increase in protein acetylation beginning at 2 h after MAKV-8 treatment, with a peak occurring between 8 and 24 h. With SAHA, we observed similar effects for acetyl-α-tubulin, but histone H4 acetylation was maintained at a later time (Fig. 3b).

**Table 3:** EC_50_ values of MAKV-8 and SAHA towards acetylated targets

Target	EC_**50**_ (μM)	
	MAKV-8	SAHA
**Acetyl α-tubulin**	12.1 ± 0.9	1.9 ± 0.8
**Acetyl histone H4**	5.9 ± 1.4	2.6 ± 0.2

Data are presented as the mean ± standard deviation of the effective concentration that induces half-maximal acetylation of protein targets (EC_50_). Values were calculated after western blot quantification from at least three independent experiments. Concentrations of compounds near the EC_50_ values have been used for subsequent experiments

### MAKV-8 displayed cytotoxic properties in CML cells

We further evaluated whether MAKV-8 treatments exert anti-CML properties. MAKV-8 inhibited the growth of CML cell lines (i.e., K-562, KBM-5, and MEG-01) starting at about 3 μM, and cell death was triggered beginning at 10 μM after 48 h of treatment (Fig. 3c). Accordingly, the replicative ability of CML cells in a 3D model was strongly reduced by MAKV-8, beginning at 5 μM in K-562 and MEG-01 cells and 10 μM in KBM-5 cells (Fig. 3d). Notably, MAKV-8 exposure also enhanced α-tubulin and histone H4 acetylation in KBM-5 and MEG-01 cells, which generalized our findings concerning MAKV-8-mediated inhibition of multiple HDAC activities in CML cells (Fig. 3e).

### MAKV-8 derivatives were less potent than their parent compound

**Fig. 4:**
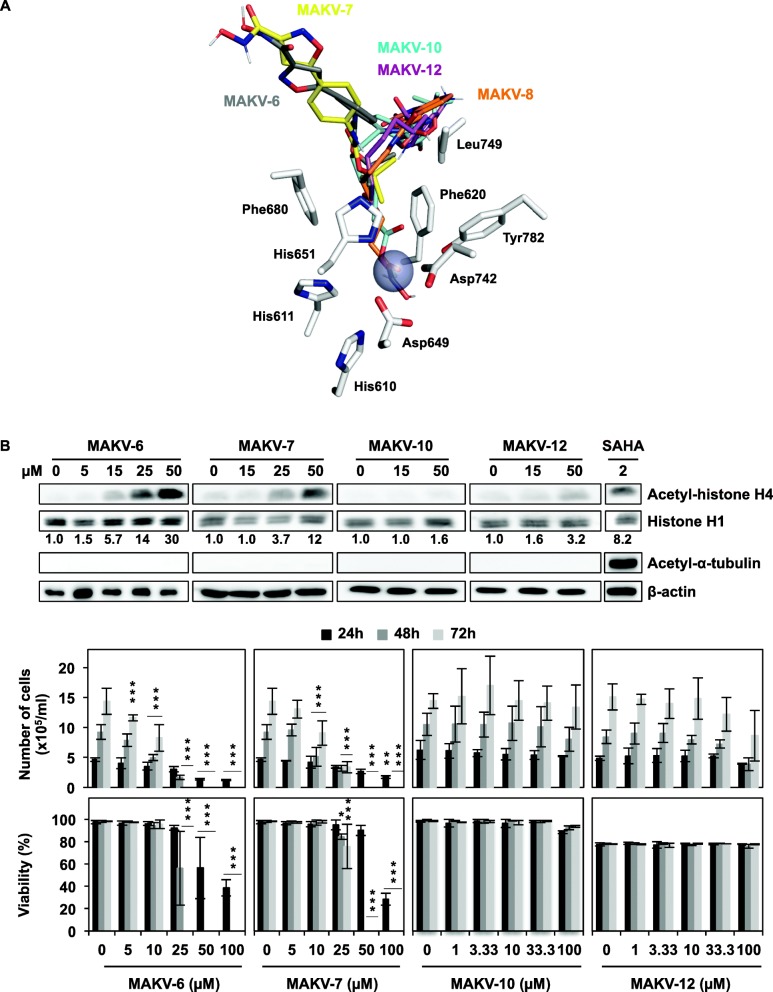
MAKV-8 derivatives display lower potency than their parent compound. (**a**) Docking poses of MAKV-8 derivatives (stick model) on HDAC6 crystal structure (white; PDB code: 5EDU). Numbered residues forming hydrophobic interactions in the binding sites (stick representation) are indicated. Zinc atom is shown as a purple sphere; nitrogen and oxygen are colored in blue and red, respectively. (**b**) Histone H4 and α-tubulin acetylation levels were assessed by western blot (upper panel), and cell proliferation and viability were evaluated (lower panel) following treatments of K-562 cells with increasing concentrations of the indicated MAKV-8 derivatives for 24h and up to 72h, respectively. β-actin and histone H1 served as loading controls for α-tubulin and histone H4, respectively. Blots are representative of three independent experiments. SAHA was used as a reference HDACi

To gain insight into the relationship between MAKV-8 structure and deacetylase-inhibiting activities, we tested the HDAC-inhibitory potential of four MAKV-8 derivatives (Fig. 1). In vitro, IC_50_ values for MAKV-6 and MAKV-7 against total HDAC activities were in the low micromolar range (Table 4), indicating that compounds were less potent than MAKV-8, whereas MAKV-10 and MAKV-12 failed to inhibit HDAC activities with concentrations up to 100 μM (Additional file 1: Figure S2). Compared to MAKV-8, docking analysis showed weaker binding affinities of the derivatives to HDAC6; MAKV-6 and MAKV-7 could not bind properly to its ligand-binding pocket, whereas MAKV-10 and MAKV-12 moderate binding did not allow suitable interactions with the zinc atom (Fig. 4a; Table 5). We confirmed these results in K-562 cells by showing that MAKV-6 and MAKV-7 increased histone H4 acetylation only at the highest concentrations and failed to increase α-tubulin acetylation, suggesting that HDACs targeting histones were preferentially inhibited, but with a much lower potency than MAKV-8. Conversely, MAKV-10 and MAKV-12 did not augment protein target acetylation levels even at the highest concentrations tested (Fig. 4b). After 48 h of treatment with MAKV-8 derivatives, we detected a reduction in K-562 cell proliferation starting at 5 μM MAKV-6 or 10 μM MAKV-7 and a decrease of cell viability starting at 25 μM MAKV-6 or MAKV-7. In contrast, neither MAKV-10 nor MAKV-12 impacted K-562 cell proliferation and viability (Fig. 4b), suggesting that MAKV-8 anti-cancer effects are associated with the inhibition of HDACs targeting histones.

**Table 4:** In vitro HDAC-inhibitory activity of MAKV-8 and derived compounds

Compounds	IC_50_ values against total HDAC activity (nM)
MAKV-6	1050 ± 220
MAKV-7	22,000 ± 25,200
MAKV-8	5.8 ± 3.8
MAKV-10	> 100,000
MAKV-12	> 100,000

Data are presented as the mean ± standard deviation of the concentration inhibiting 50% (IC_50_) of the HDAC activity. Values were calculated from at least three independent experiments or two independent experiments for MAKV-10 and MAKV-12

*HDAC* histone deacetylase

**Table 5:** Qualitative molecular docking of MAKV-8 derivatives against HDAC6

Compounds	Binding affinity (kcal/mol)^a^
MAKV-6	− 5.9
MAKV-7	− 6.4
MAKV-8	− 7.2
MAKV-10	− 6.9
MAKV-12	− 6.1

^a^Binding affinity energy values for Protein Data Bank (PDB) code 5EDU were calculated using AutoDock Vina program

### MAKV-8 induced cell cycle arrest and apoptotic cell death in CML cells

**Fig. 5:**
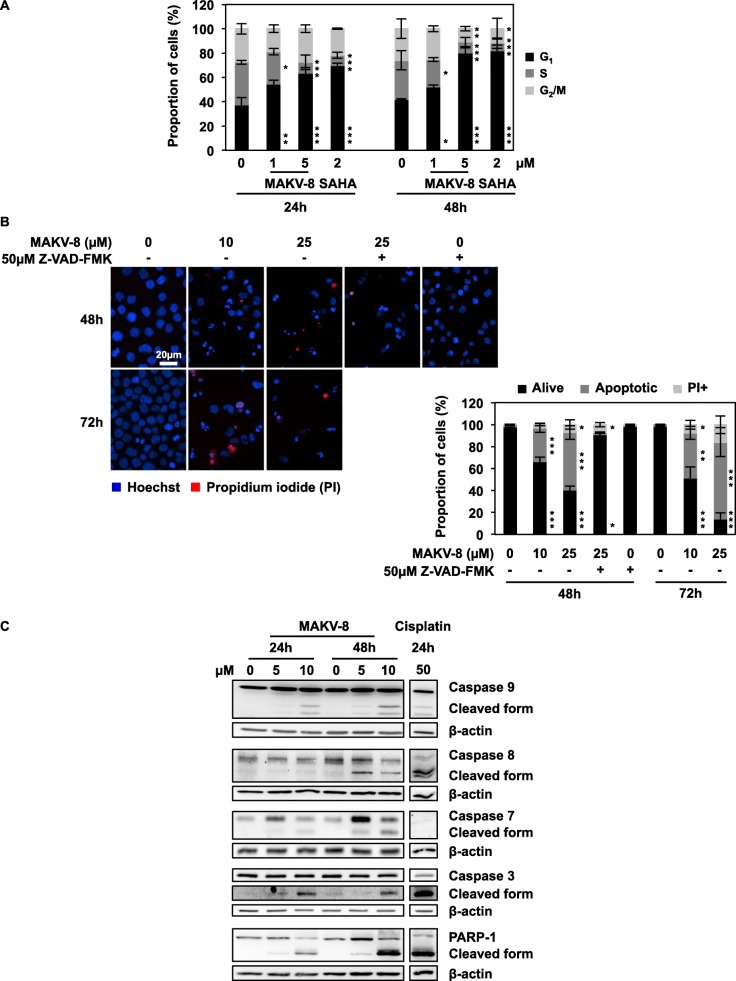
Treatment with MAKV-8 leads to cell cycle arrest and apoptotic cell death. K-562 cells were treated with MAKV-8 at the indicated time points and concentrations, followed by analyses of (**a**) cell cycle distribution using a range of subtoxic cytostatic MAKV-8 concentrations to focus only on aspects of cell cycle modulation, (**b**) nuclear morphology, and (**c**) caspase and PARP-1 activation. (**b**) Representative pictures of cells stained with Hoechst in blue and propidium iodide (PI) in red (upper left panel) and corresponding quantifications (lower right panel) from three independent experiments are provided. Where indicated, cells were pre-incubated for 1h with the pan-caspase inhibitor z-VAD-FMK. Cisplatin was used as a positive control for caspase and PARP-1 cleavage. Blots used β-actin as the loading control and are representative of three independent experiments. SAHA was used as a reference HDACi

Next, we further characterized the cytostatic and cytotoxic properties of MAKV-8 in CML cells. Since MAKV-8 treatment hampered CML cell proliferation, we studied its effects on cell cycle distribution, which revealed a time-dependent accumulation of cells in the G1 phase, which was comparable to results obtained with SAHA (Fig. 5a).

Nuclear morphology analyses showed that MAKV-8 mainly triggered a time- and dose-dependent increase in apoptotic cells, which was completely prevented upon treatment with the pan-caspase inhibitor z-VAD-FMK (Fig. 5b). In addition, cleavage of pro-caspases 3, 7, 8, and 9 and poly (ADP-ribose) polymerase (PARP)-1 was consistent with caspase-dependent apoptosis induction (Fig. 5c).

### MAKV-8 triggered ER stress, autophagic flux, and DNA damage

**Fig. 6:**
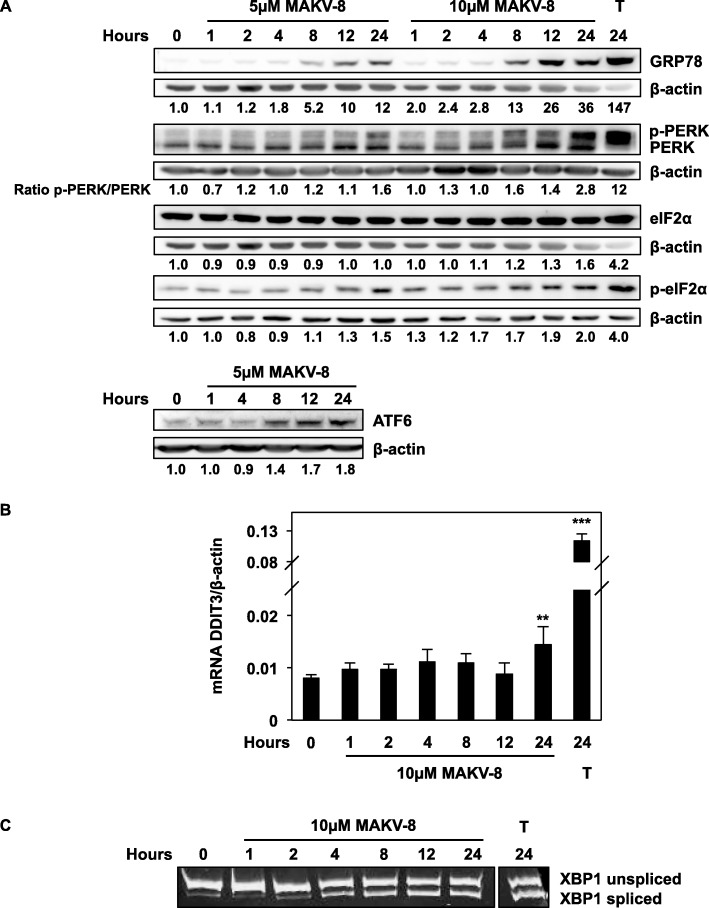
MAKV-8 treatment induces ER stress. K-562 cells were treated with the indicated concentrations of MAKV-8 at the indicated time points unless otherwise stated. (**a**) The expression levels of UPR-associated proteins, such as the ER stress marker GRP78, were assessed by western blot using β-actin as a loading control. (**b**) DDIT3 mRNA expression levels were quantified by real-time PCR and normalized to β-actin mRNA levels. (**c**) End-point analysis of XBP1 mRNA splicing. Thapsigargin (T, 4 µM) was used as a positive control for ER stress induction. All pictures are representative of three independent experiments

Since the inhibition of cell cycle progression could result from endoplasmic reticulum (ER) stress-induced unfolded protein response (UPR) activation [16], we analyzed 78 kDa glucose-regulated protein (GRP78) expression levels in K-562 cells upon treatments with MAKV-8 compared to thapsigargin. GRP78 expression was upregulated after 8 h of treatment and accompanied by increased ATF6 expression, as well as phosphorylated protein kinase RNA-like ER kinase (PERK) and eukaryotic initiation factor (eIF)2α levels (Fig. 6a). Accordingly, we also observed enhanced DNA damage-inducible transcript (DDIT)3 mRNA expression after 24 h (Fig. 6b) and X-box binding protein (XBP)1 mRNA splicing after 4 h (Fig. 6c) upon treatment with 10 μM MAKV-8. Altogether, results demonstrate that MAKV-8 activates the UPR signaling.

**Fig. 7:**
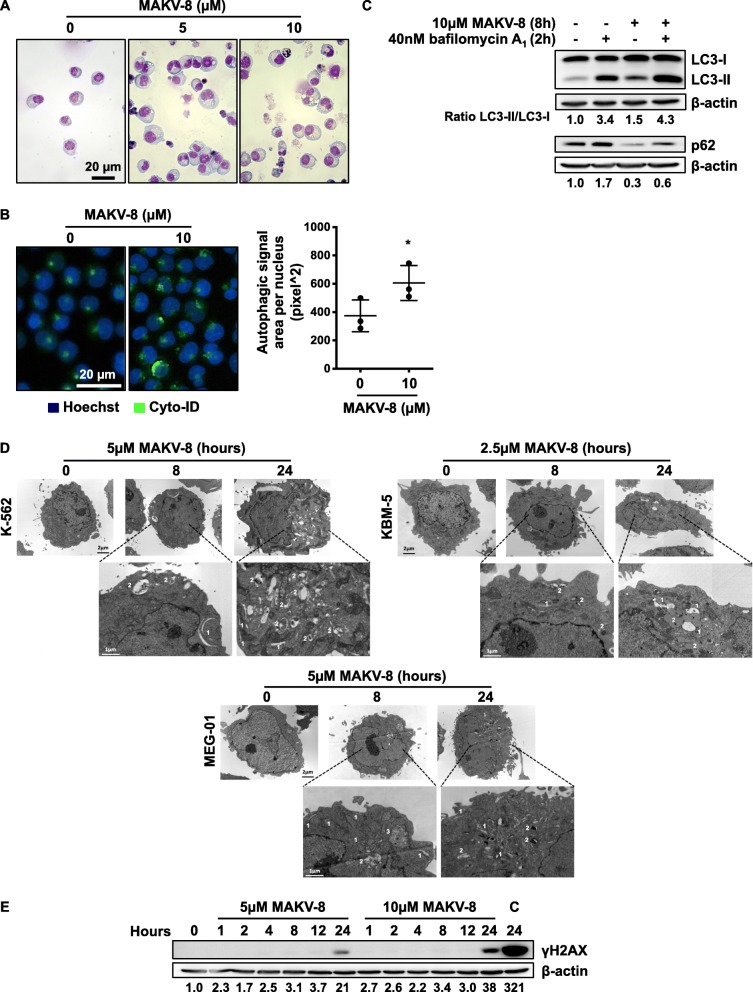
MAKV-8 treatment triggers autophagy and double strand breaks. K-562 cells were treated with the indicated concentrations of MAKV-8 at the indicated time points unless otherwise stated. (**a**) Cell morphology was analyzed after 48h of treatment using modified GIEMSA staining, and pictures were acquired by bright-field microscopy. (**b**) The appearance of autophagosome-related vesicles was quantified in cells treated with MAKV-8 for 8h. Representative pictures of cells stained with Hoechst in blue and Cyto-ID in green (left panel) and corresponding quantifications (right panel) from three independent experiments are provided. (**c**) After 8h of treatment, the conversion of LC3-I to LC3-II and expression of p62, two autophagic markers, were evaluated by western blot. Where indicated, bafilomycin A_1_ was added 2h before the end of treatment. (**d**) Representative images of electron microscopy analysis in indicated CML cell line: (1) phagophores and (2) autophagolysosomes. (**e**) The expression level of γH2AX, the earliest marker for DNA damage localized at double strand breaks, was assessed by western blot. Cisplatin (C, 50 µM) was used as a positive control for double strand break induction. Blots used β-actin as the loading control, and pictures are representative of three independent experiments

Modulations of the autophagic machinery, which can be activated by the UPR pathways [17], may contribute to HDACi anti-cancer effects [18]. Cell morphology analyses revealed that, unlike control cells, MAKV-8-treated cells displayed a swollen cytoplasm enriched with numerous vacuoles of various sizes (Fig. 7a). Additionally, Cyto-ID®-stained cell quantification showed that the autophagic signal area per nucleus was about 1.5 times higher in MAKV-8-treated cells compared to controls (Fig. 7b). Furthermore, MAKV-8 treatment stimulated microtubule-associated protein 1 light chain (LC)3-I to LC3-II conversion and reduced p62/sequestosome (SQSTM)1 expression levels. Upon addition of late-phase autophagy inhibitor bafilomycin A1, LC3-II and p62/SQSTM1 accumulation was further enhanced, suggesting that MAKV-8 induced autophagy (Fig. 7c). Accordingly, the study of cellular structures by transmission electron microscopy showed a more extensive autophagocytic vacuolization in MAKV-8-treated cells compared to untreated cells (Fig. 7d).

HDACi treatments reportedly cause DNA damage, including double strand breaks, which partly underlies apoptosis induction [19]. Accordingly, we showed that 5 μM MAKV-8 enhanced H2AX phosphorylation (γH2AX) levels after 24 h; this effect was more pronounced with 10 μM MAKV-8 (Fig. 7e), although less important than that observed with cisplatin [20].

### Modulation of HDAC expression profiles in CML patients

**Fig. 8:**
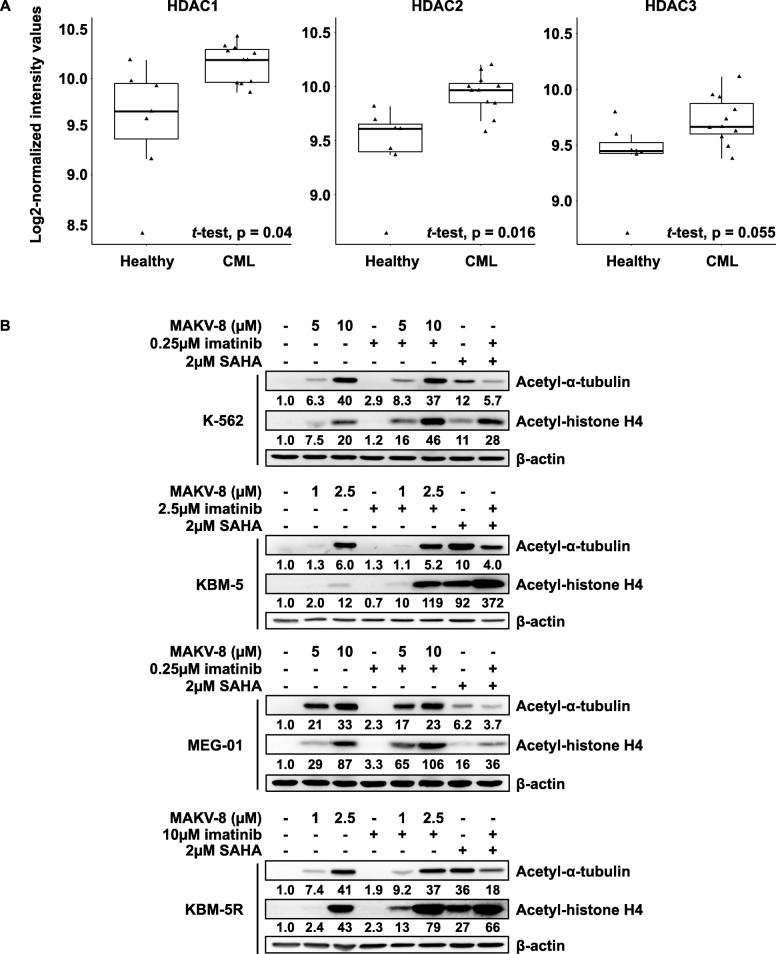
Treating CML cells with imatinib in combination with pan-HDACis is a promising therapeutic approach. (**a**) Boxplots including outliers illustrating fold-change (log2) of HDAC1, HDAC2 and HDAC3 mRNA expression levels in CD34+CD38- stem cells isolated from healthy (n=7) and CML (n=11) patients (represented by triangles). (**b**) CML cells were treated with the indicated concentrations of imatinib alone or in combination with MAKV-8. After 24h-incubations, α-tubulin and histone H4 acetylation levels were assessed by western blot, with β-actin and histone H1 as loading controls, respectively. Blots are representative of three independent experiments. SAHA was used as a reference HDACi

Despite the outstanding therapeutic results obtained with TKis in CML, the occurrence of imatinib resistance in over 30% of CML patients necessitates the discovery of additional therapeutic approaches. Interestingly, transcriptomic analyses of CML patient data revealed that HDAC1 and HDAC2 mRNA expression levels were significantly upregulated and associated with a trend towards increased HDAC3 mRNA levels in LSCs compared to healthy stem cells (HSCs) (Fig. 8a). The importance of HDAC1 and HDAC2 in tumor cell survival provides a good rational for treating CML cells with imatinib in combination with pan-HDACis [21].

### MAKV-8 combined with imatinib displayed synergistic pro-apoptotic activity in imatinib-sensitive and imatinib-resistant CML cells

Considering computational docking results, we investigated the anti-leukemic potential of co-treatments with subtoxic concentrations of MAKV-8 and imatinib in imatinib-sensitive and imatinib-resistant CML cells. First, we tested whether treatment with imatinib affected MAKV-8-mediated HDAC inhibition. In all CML cells, α-tubulin acetylation was similarly induced after a 24-h exposure to MAKV-8-imatinib co-treatment compared to MAKV-8 treatment alone, whereas acetylated histone H4 levels were further enhanced. In contrast, cells treated with SAHA-imatinib displayed decreased and increased levels of acetylated α-tubulin and histone H4, respectively, compared to SAHA alone (Fig. 8b).

**Fig. 9:**
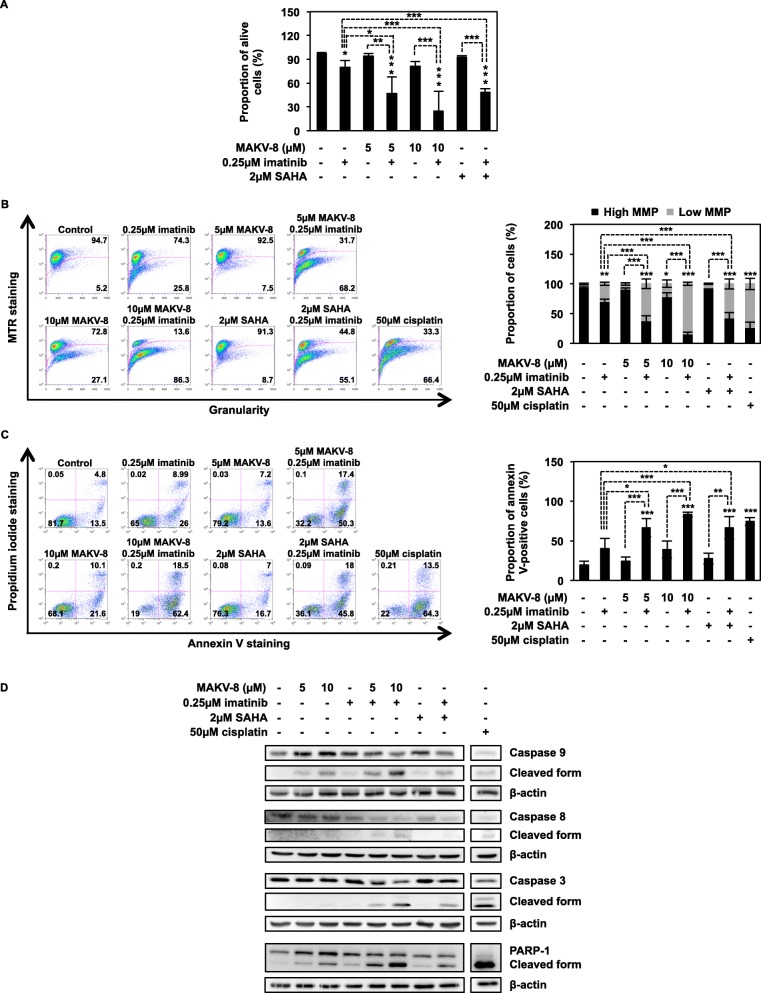
The HDACi MAKV-8 combined with imatinib induces synergistic anti-cancer activity in K-562 cells. Cells were treated with the indicated concentrations of imatinib alone or in combination with MAKV-8. (**a**) Nuclear morphology, (**b**) phosphatidylserine exposure, and (**c**) mitochondrial membrane potential (MMP) were analyzed in K-562 cells treated for 48h. Representative dot plots (left panel) and corresponding quantifications (right panel) from three independent experiments are provided. (**d**) Caspase and PARP-1 cleavages were analyzed by western blot in K-562 cells treated for 24h, using β-actin as the loading control. Cisplatin was used as a positive control for apoptosis induction and MMP disruption. Blots and pictures are representative of three independent experiments. SAHA was used as a reference HDACi

We then evaluated the effect of combinations on CML cell viability. In K-562 cells, MAKV-8-imatinib-mediated reduction in viability was greater than that induced by compounds alone, with a 75%-decrease in living cells after treatment with 10 μM MAKV-8 combined with imatinib (Fig. 9a). Combination index (CI) values below 1 indicated synergism for each MAKV-8 concentration combined with imatinib, with the highest MAKV-8 concentration conferring the maximal effect (Table 6). Additionally, MAKV-8-imatinib co-treatment triggered mitochondrial membrane potential (MMP) loss and increased Annexin V-positive cell proportion, reaching approximately 83% of cells displaying low MMP and Annexin V positivity, respectively, after co-treatments with 10 μM MAKV-8 compared to 7 and 20% in untreated cells (Fig. 9b, c). Such co-treatment also caused a stronger cleavage of caspases 3, 8, 9, and PARP-1, indicating more important apoptosis induction than observed with single treatments (Fig. 9d).

**Table 6:** Combination index (CI) values for treatments with combined MAKV-8-imatinib in imatinib-sensitive and imatinib-resistant CML cells

Cell line	MAKV-8 (μM)	Imatinib (μM)	CI (48 h)^**a**^
**K-562**	5	0.25	0.42 ± 0.13
	10	0.25	0.28 ± 0.14
**KBM-5**	1	2.5	0.46 ± 0.06
	2.5	2.5	0.05 ± 0.03
**MEG-01**	5	0.25	0.77 ± 0.16
	10	0.25	0.37 ± 0.07
**KBM-5R**	1	10	1.42 ± 0.58
	2.5	10	0.78 ± 0.16

^a^CI values correspond to the mean ± standard deviation of three independent Hoechst-propidium iodide staining experiments

*CML* chronic myeloid leukemia

**Fig. 10:**
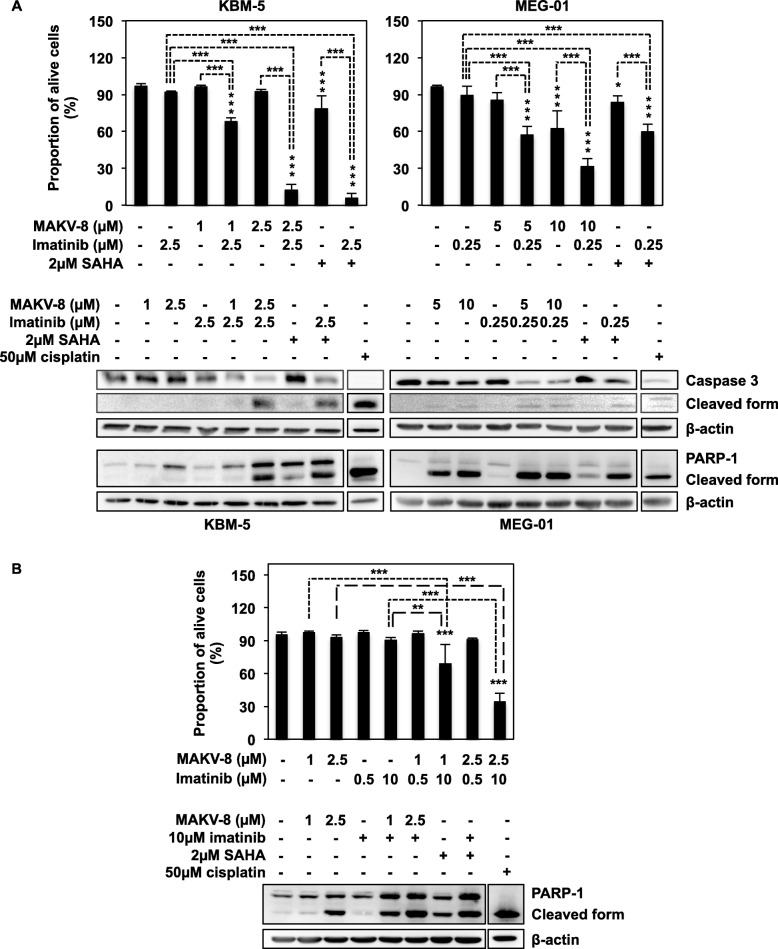
MAKV-8 combined with imatinib induces synergistic anti-cancer activity in imatinib-sensitive and -resistant CML cells. CML cells were treated with the indicated concentrations of imatinib alone or in combination with MAKV-8. (**a**) Nuclear morphology (upper panel) and cleavage of caspase 3 and PARP-1 (lower panel) were studied in KBM-5 and MEG-01 cells treated for 48 and 24h, respectively. (**b**) Nuclear morphology (upper panel) and PARP-1 cleavage (lower panel) were evaluated in KBM-5R cells treated for 48 and 24h, respectively. Caspase and PARP-1 cleavages were assessed by western blot using β-actin as the loading control. Cisplatin was used as a positive control. Blots are representative of three independent experiments. SAHA was used as a reference HDACi

We generalized our findings by showing that MAKV-8-imatinib combination also had a synergistic effect on KBM-5 and MEG-01 cell viability, with a reduction of 88 and 69% of living cells, respectively, following co-treatments with the highest MAKV-8 concentration (Fig. 10a, upper panel, Table 6). Additionally, caspase 3 and PARP-1 cleavage highlighted a greater induction of apoptosis in MAKV-8-imatinib co-treated KBM-5 and MEG-01 cells (Fig. 10a, lower panel). Comparatively, co-treatments with 2 μM SAHA presented similar results to those obtained after co-treatment with the lowest MAKV-8 concentration in all CML cells (Figs. 9 and 10a).

In imatinib-resistant KBM-5 (KBM-5R) cells, which began to die in response to a dose between 10 and 25 μM imatinib (Additional file 1: Figure S3), the decrease in cell viability was further enhanced by co-treatments compared to treatments with either drug alone, and a synergistic loss of 65% of living cells occurred after a combination with 2.5 μM MAKV-8 and 10 μM imatinib (Fig. 10b, upper panel; Table 6). Accordingly, PARP-1 cleavage, which indicated the activation of apoptotic pathways, was stronger in co-treated KBM-5R cells (Fig. 10b, lower panel).

### Imatinib-MAKV-8 co-treatment induced differential toxicity in healthy cell models compared to CML cells

**Fig. 11:**
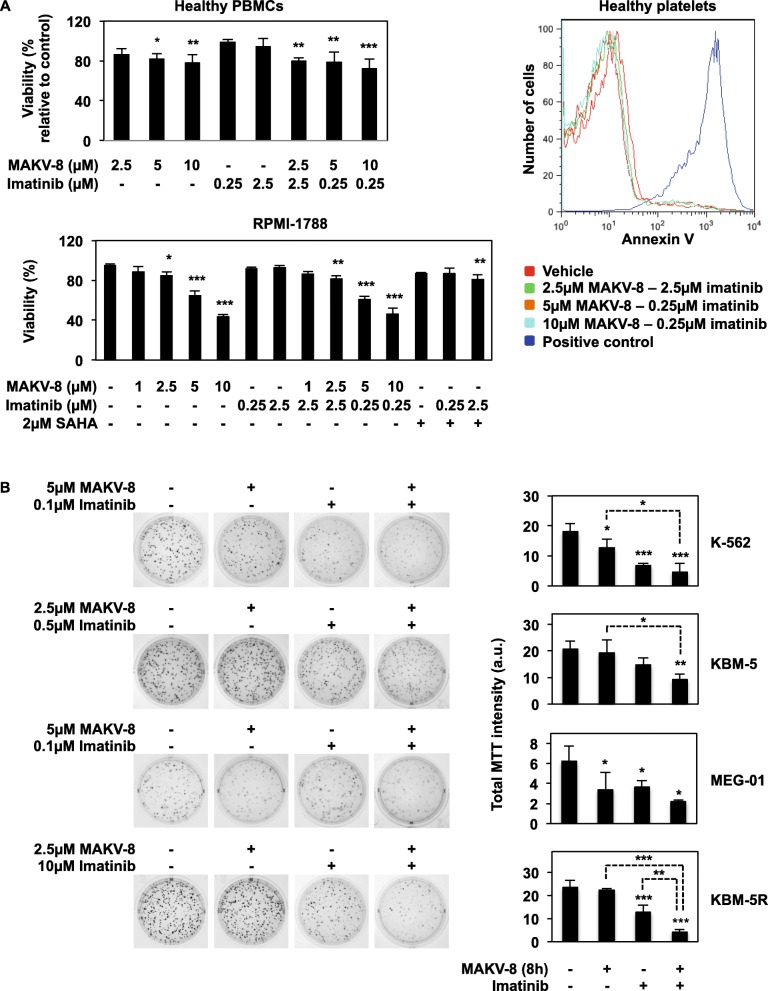
MAKV-8 combined with imatinib displays a differential toxicity in healthy cells compared to CML cells. CML and healthy cells were treated with the indicated concentrations of imatinib alone or in combination with MAKV-8. (**a**) Healthy cell models were treated for 48h. Cell viability was assessed based on the Trypan Blue exclusion method for PBMCs, by flow cytometry after Annexin V staining for platelets, and nuclear morphology was examined in RPMI-1788 cells. SAHA was used as a reference HDACi. (**b**) CML cells were pre-treated with MAKV-8 for 8h and then grown in semisolid methylcellulose medium in the presence of imatinib. After 10-day incubations, cell colony-forming capacity was scored after MTT addition. Representative pictures (left panel) and corresponding quantifications (right panel) from three independent experiments are provided

Next, we evaluated the effect of MAKV-8-imatinib co-treatments on the viability of healthy cell models. In peripheral blood mononuclear cells (PBMCs), we observed a moderate effect with MAKV-8 treatment either alone or in combination, with a maximum decrease of 30% of viable cells after 48 h, whereas imatinib alone failed to trigger any cell mortality (Fig. 11a, upper panel; Additional file 1: Figure S4). These results were comparable to those of SAHA (Additional file 1: Figure S4). Although RPMI-1788 cells underwent a reduction of about 55% of living cells following a 48-h treatment with 10 μM MAKV-8, the decrease in cell viability was not further enhanced upon addition of imatinib (Fig. 11a, lower panel). Consequently, the ratio of cell death induced by MAKV-8-imatinib co-treatments in cancer versus normal cells attested of a stronger toxicity against cancer cells (Table 7). Finally, MAKV-8 neither alone nor in co-treatments elicited any significant cytotoxicity in platelets (Fig. 11a, right panel; Additional file 1: Figure S4). Altogether, combination of MAKV-8 with imatinib displayed a promising safety profile for healthy cells.

**Table 7:** Selectivity ratio of MAKV-8-imatinib co-treatment for cancer cells versus healthy models

CML cells	Selectivity ratio	
	PBMCs	RPMI-1788
**K-562**	5.8	3.9
**KBM-5**	8.5	9.2
**MEG-01**	2.2	1.5

Values were calculated from the percentage of cancer cell death versus healthy cell death from three independent experiments

*PBMCs* peripheral blood mononuclear cells

Finally, we investigated the ability of CML cell lines pre-treated with MAKV-8 for 8 h to form colonies in the presence of experimentally selected imatinib concentrations (Additional file 1: Figure S5). The combinations exerted more potent effects on reducing colony formation than agents alone in both imatinib-sensitive and imatinib-resistant CML cells (Fig. 11b).

### Beclin-1 knockdown partially prevented MAKV-8-imatinib combination-induced apoptosis

**Fig. 12:**
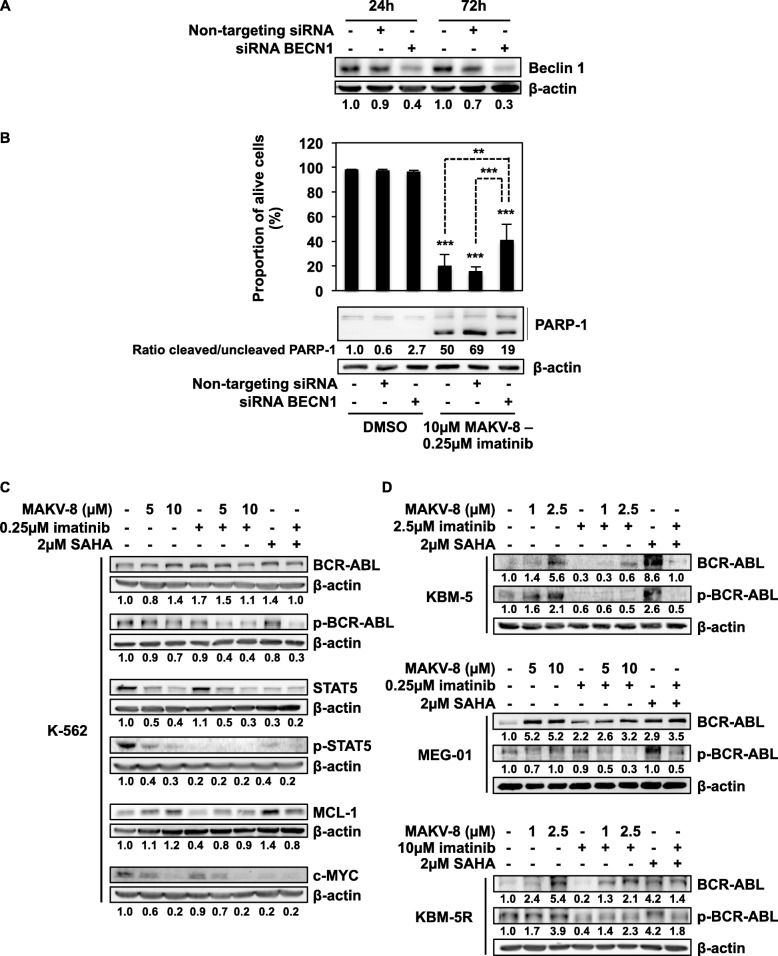
Altered BCR-ABL signaling and autophagy induction are associated with MAKV-8-imatinib anti-cancer properties. CML cells were treated with the indicated concentrations of imatinib alone or in combination with MAKV-8. (**a**, **b**) K-562 cells were transfected with or without the indicated siRNA, then (**a**) the expression level of beclin-1, a protein involved in initiating the autophagic flux, was assessed by western blot 24 and 72h post-transfection, and (**b**) nuclear morphology (upper panel) and PARP-1 cleavage (lower panel) were analyzed in cells treated for 24 and 48h, respectively. The ratio between the cleaved and uncleaved forms of PARP-1 was determined based on western blot quantification. (**c**, **d**) Protein expression and phosphorylation levels were assessed by western blot in cells treated for 24h. Blots used β-actin as a loading control and are representative of three independent experiments. SAHA was used as a reference HDACi

Since MAKV-8 treatments induced autophagy, we tested whether this process would be implicated in co-treatment-induced synergistic cell death. We knocked down the gene coding for beclin-1 (*BECN1*) (Fig. 12a) and observed after 48 h of MAKV-8-imatinib treatment that the proportion of viable K-562 cells was significantly increased from 15 and 20% in cells transfected with or without non-targeting small interfering RNA (siRNA), respectively, to 40% in *BECN1*-silenced cells (Fig. 12b, upper panel). Finally, decreased PARP-1 cleavage in cells transfected with siRNA targeting BECN1 further confirmed reduced apoptotic rate (Fig. 12b, lower panel).

### MAKV-8 and imatinib co-treatment reduced BCR-ABL-related pathways

BCR-ABL-related signaling pathways result in CML cell growth and survival [22]. Accordingly, we examined the effects of MAKV-8-imatinib co-treatment on the expression and phosphorylation of BCR-ABL and downstream targets in K-562 cells. Although such combination did not impact BCR-ABL expression, it led to a drastic decrease in its phosphorylation accompanied by a similar effect on signal transducer and activator of transcription (STAT)5 phosphorylation. Notably, MAKV-8 further downregulated STAT5 protein levels and provoked a striking decrease in c-MYC expression, which was maintained by co-treatment with imatinib. Conversely, myeloid cell leukemia (MCL)-1 expression was not markedly impacted by the combination due to oppositional effects exhibited by each drug (Fig. 12c). Despite a MAKV-8-mediated upregulation in BCR-ABL expression, treatment with MAKV-8 and imatinib decreased BCR-ABL phosphorylation in KBM-5, MEG-01, and KBM5-R cells (Fig. 12d). In all CML cell lines, treatments with SAHA, alone or in combination with imatinib, exhibited results comparable to that of the highest MAKV-8 concentration (Fig. 12c, d).

### MAKV-8 and imatinib co-treatment reduced the LSC population and inhibited CML cell growth in vivo

**Fig. 13:**
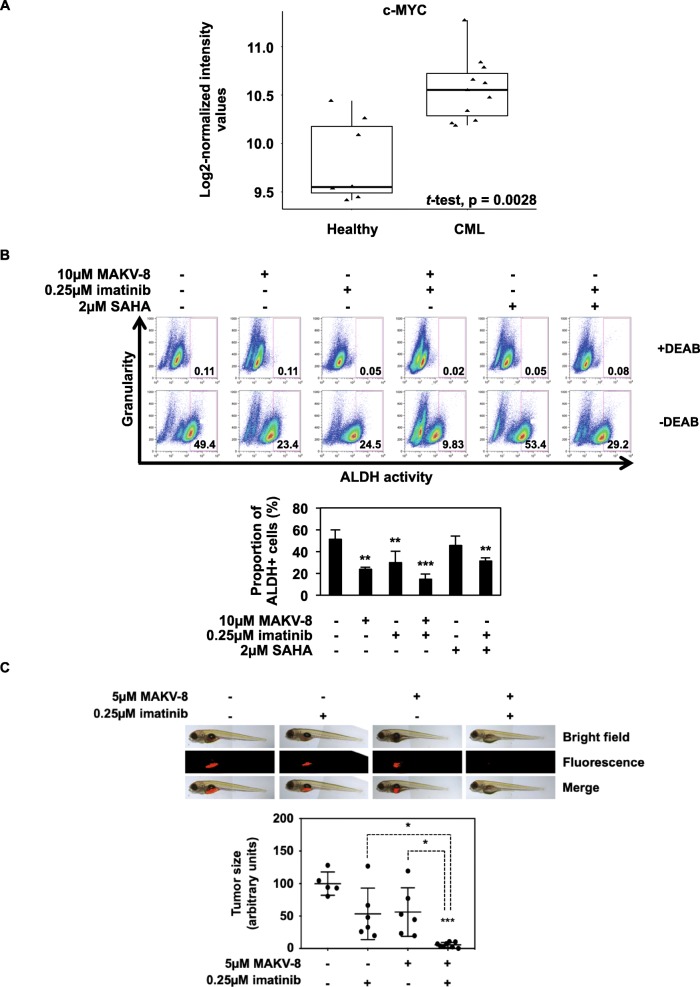
MAKV-8-imatinib combination reduces cancer stem cell population. CML cells were treated with the indicated concentrations of imatinib alone or in combination with MAKV-8. (**a**) Boxplots illustrating fold-change (log2) of c-MYC mRNA expression in CD34+CD38- stem cells isolated from healthy (n=7) and CML (n=11) patients (represented by triangles). (**b**) Analysis of aldehyde dehydrogenase (ALDH) activity in K-562 cells cultured for 24h and known to present a substantial proportion of cells with cancer stem-like characteristics. Elevated ALDH activity is an established marker for the identification of hematopoietic stem cells. The ALDH inhibitor diethylaminobenzaldehyde (DEAB) was used to distinguish cell subpopulations with low and high ALDH activity. Representative dot plots where the percentage of ALDH+ cells is indicated (upper panel) and corresponding quantifications (lower panel) representative of three independent experiments are presented. SAHA was used as a reference HDACi. (**c**) K-562 cells were treated for 24h, fluorescently labeled, and then injected into the zebrafish yolk sac. Three days post-injection, pictures of 5 to 8 fishes (one representative set of pictures is presented) were taken, and the fluorescence intensity was quantified

The oncogenic transcription factor c-MYC reportedly plays an important role in LSC survival, which is implicated in TKi resistance and relapse in CML patients [23]. Moreover, bioinformatic analysis revealed that c-MYC mRNA expression levels were significantly upregulated in LSCs versus HSCs (Fig. 13a).

Since MAKV-8 strongly reduced c-MYC expression (Fig. 12c), we further evaluated whether MAKV-8 treatment, with and without imatinib, could decrease the aldehyde dehydrogenase (ALDH)^+^ LSC fraction in K-562 cells. The ALDH^+^ cell percentage was reduced from 50% in untreated cells to 24 and 30% in MAKV-8- and imatinib-treated cells, respectively, and was further decreased to 15% after MAKV-8-imatinib co-treatment. In contrast, SAHA failed to reduce the ALDH^+^ cell proportion. When cells were co-treated with SAHA and imatinib, the percentage of ALDH^+^ cells decreased to 30%, indicating the MAKV-8-imatinib combination was more efficient to reduce LSC population (Fig. 13b).

We finally tested our findings in an in vivo setting and demonstrated that combining MAKV-8 with imatinib fully abrogated tumor formation in zebrafish xenografted with K-562 cells, whereas single agents were unable to significantly reduce tumor volumes (Fig. 13c; Additional file 1: Figure S6).

## Discussion

In vitro, MAKV-8 acts as 10 times more potent pan-HDACi than SAHA. Accordingly, docking studies suggest efficient MAKV-8 interactions with the ligand-binding pockets of all tested HDACs, with higher binding affinity in comparison to SAHA. Interestingly, EC_50_ values for MAKV-8 and SAHA towards acetylated protein targets, as well as the different modulations of compounds-mediated HDAC inhibition upon imatinib addition suggest distinct HDAC-inhibitory properties. Compared to SAHA, MAKV-8-mediated acetylation of specific histone and non-histone proteins may result in improved HDACi-related anti-cancer activities. Despite a fairly good overall in silico profile, some structural modifications could be necessary to improve MAKV-8 drug-likeness parameters. Nevertheless, the importance of the hydroxamate group and the linker chain length for HDAC-inhibitory activities was confirmed by the results obtained with MAKV-8 derivatives [15]. Notably, in silico predictions should be critically considered due to potentially different pharmacokinetic properties of compounds in vivo. Collectively, MAKV-8 appears to be an attractive drug candidate for further consideration in CML therapies, especially considering that the inhibition of HDAC1 and HDAC2, upregulated in LSCs versus HSCs from patients, strongly impacts the transcription of proteins essential for tumor cell survival [21].

MAKV-8 treatment triggers ER stress, as evidenced by the upregulation of proteins related to UPR. Since HDACs have been described to modulate GRP78 acetylation [24], MAKV-8-mediated HDAC inhibition could result in GRP78 acetylation and selective UPR activation, as similarly reported for other HDACi [25]. Furthermore, MAKV-8-mediated inhibition of HDAC6, which is important for misfolded protein clearance, could thus lead to the accumulation of protein aggregates and ER stress induction [26]. Simultaneously to ER stress, MAKV-8 induces autophagy. Pan-HDACi have been shown to promote the initiation and block the maturation phases of autophagy by inhibiting class I–IIa and class IIb HDACs, respectively [27]. Autophagy may also participate in imatinib sensitization, as downregulation of pro-autophagic beclin-1 expression partly prevents co-treatment-induced cell death. Conversely, one study showed that impairing autophagy significantly enhanced SAHA anti-cancer activity [28]. Finally, MAKV-8 treatment weakly induces the appearance of double strand breaks at a time where no cell death is observed. Since H2AX phosphorylation does not occur in concomitance with histone acetylation, as previously reported [29], MAKV-8-mediated accumulation of excessive DNA damage may result from ER stress and/or autophagy, and this accumulation could lead to apoptosis activation [30]. Altogether, our work does not exclude that MAKV-8-related mechanisms could be implicated in its cytotoxic effects, but further studies are required. Importantly, the lack of differential toxicity in CML versus healthy cells following MAKV-8 treatment strongly limits its use as a monotherapy. Nevertheless, the treatment of cancer patients with single agents is increasingly replaced by combination therapies to prevent adaptive response and acquired drug resistance [31].

In imatinib-sensitive and imatinib-resistant BCR-ABL-positive CML cells, synergistic apoptotic cell death is triggered upon MAKV-8-imatinib co-treatment. Accordingly, concomitant treatment with SAHA and TKi has previously been reported to synergistically induce apoptosis in CML cell lines [13, 14]. Furthermore, MAKV-8 combined with imatinib also impaired tumor growth of xenografted CML cells in zebrafish, which is considered as an appropriate vertebrate model organism owing to the high percentage of orthologous genes between zebrafish and humans [32]. Notably, moderate cytotoxicity was observed in healthy models exposed to the same co-treatment. A weak toxicity towards normal cells compared to cancerous cells has also been described for many other HDACi [33, 34]. Collectively, MAKV-8 could provide a non-toxic strategy for overcoming imatinib resistance, hence providing a rational basis to further study the potency of MAKV-8-imatinib combination for CML therapy.

Mechanistically, lowered BCR-ABL kinase activity is observed after MAKV-8-imatinib co-treatment, which most likely explains the reduction in STAT5 phosphorylation, as well as the drop in c-MYC and MCL-1 expression. Previous reports indicate that SAHA downregulates BCR-ABL mRNA and protein levels in CML cells [13], which could be implicated in synergistic anti-leukemic interactions involving TKis [14]. By inhibiting HDAC6, pan-HDACis could disrupt the association of HSP90α with BCR-ABL, provoking its poly-ubiquitination and proteasomal degradation [35]. Surprisingly, MAKV-8 rather augments BCR-ABL expression in our cell models, implying the involvement of other regulatory mechanisms. Furthermore, downstream BCR-ABL targets play a critical role in CML pathogenesis [36–38]. Similar to our observations, HDACi-mediated potentiation of TKi cytotoxicity has been related to STAT5 inhibition in BCR-ABL-positive cells [39]. As previously described, HDACi-related apoptosis induction in CML cells could be enhanced by imatinib-mediated downregulation of anti-apoptotic MCL-1 expression, which was upregulated by MAKV-8 [40]. In addition, c-MYC has been recently reported as an important target for selectively eliminating CML LSCs [41]. Accordingly, human LSCs displayed increased c-MYC mRNA levels compared to HSCs. Besides lowering c-MYC expression, MAKV-8 treatment potently decreased the ALDH^+^ cell proportion, which was further enhanced upon co-treatment with imatinib. Altogether, combining imatinib with MAKV-8, which targets key hematopoietic stem cell molecular effectors, may represent an effective strategy to overcome LSC resistance to TKis, thereby offering the opportunity to improve disease outcomes for CML patients.

## Conclusions

We found that compound MAKV-8 acted as a potent pan-HDACi in vitro and in various CML cell lines. Furthermore, MAKV-8 in combination with imatinib displayed promising anti-cancer properties in imatinib-sensitive and imatinib-resistant BCR-ABL-positive CML cells, whereas only a very moderate toxicity was noted in healthy cell models exposed to the same co-treatment. Mechanistically, the combination MAKV-8-imatinib reduced BCR-ABL expression and phosphorylation, as well as the expression of downstream targets playing a critical role in CML proliferation and survival. In addition, such therapeutic approach effectively decreased LSC population. Altogether, the present findings suggest that treatment with MAKV-8 contributes to a strong sensitization of imatinib-sensitive and imatinib-resistant CML cells including LSCs towards imatinib cytotoxicity, hence providing a rational basis to further study the potency of MAKV-8 and imatinib as a combination therapy against CML.

## Methods

### Cell culture and reagents

The human CML cell lines K-562 (DSMZ Cat# ACC-10, RRID: CVCL_0004) and MEG-01 (DSMZ Cat# ACC-364, RRID: CVCL_0425) were purchased from Deutsche Sammlung für Mikroorganismen und Zellkulturen (Braunschweig, Germany). Human CML KBM-5 cells were kindly provided by Dr. Bharat B. Aggarwal. Imatinib-resistant KBM-5 (KBM-5R) cells were established as previously described [42]. The K-562, MEG-01, and KBM-5 cell lines have been authenticated in 2014 using the LGC Standards Cell Line Authentication service (Teddington, UK). All cells were maintained at 37 °C in a humid atmosphere and 5% CO_2_ in a culture medium supplemented with 10% heat-inactivated fetal calf serum (FCS; BioWhittaker ®, Lonza, Verviers, Belgium) and 1% (v/v) antibiotic (streptomycin and penicillin) and antimycotic (BioWhittaker®). K-562 and MEG-01 cells were cultured in RPMI 1640 medium (BioWhittaker®). KBM-5 and KBM-5R cells were cultured in IMDM medium (BioWhittaker®).

PBMCs were isolated from the blood of healthy adult human donors obtained from the Red Cross (Luxembourg, Luxembourg) and purified as previously reported [43]. Human RPMI-1788 cells (ATCC Cat# CCL-156, RRID: CVCL_2710), which were derived from B lymphocytes, were purchased in 2017 from the American Type Culture Collection (Manassas, Virginia, USA). Platelets from healthy adult human donors were provided by the Red Cross. All healthy models were cultured at 37 °C in a humid atmosphere and 5% CO_2_ in RPMI 1640 medium (BioWhittaker®) supplemented with 0, 10, or 20% heat-inactivated FCS (BioWhittaker®) for platelets, PBMCs, and RPMI-1788 cells, respectively, and each containing 1% (v/v) antibiotic-antimycotic (BioWhittaker®).

All cell lines have been monthly tested for mycoplasma contamination.

Compound MAKV-8 was synthetized as previously described by Kozikowski et al. (compound 3) [15], and compounds MAKV-6, MAKV-7, MAKV-10, and MAKV-12 were derived from the reported synthetic protocol as shown in Additional file 1: Figure S7. The following additional compounds were used in this study: cisplatin in saline solution from Teva Pharmaceutical Industries Ltd. (Wilrijk, Belgium), imatinib and thapsigargin from Sigma-Aldrich (Bornem, Belgium), SAHA from Cayman Bio-connect (Huissen, Netherlands), and z-VAD-FMK from Millipore (Merck, Brussels, Belgium). Except for cisplatin, all compounds were dissolved in dimethylsulfoxide (DMSO).

### Computational analysis of public chronic myeloid leukemia datasets

The gene expression microarray datasets E-MTAB-2581 [44] and GSE97562 [45] were downloaded from the ArrayExpress database [46] and the Gene Expression Omnibus repository, respectively. Datasets were normalized using the Robust Multichip Average algorithm from the oligo R package (version 1.48.0) [47] and batch corrected using the function *removeBatchEffect* from the limma R package (version 3.40.2) [48]. The *ggboxplot* function from the ggpubr package (version 0.2.1) [49] was used to draw the boxplots in R 3.6.0 [50] and RStudio [51].

### In vitro HDAC activity assay

In vitro HDAC activities were measured as previously described [52, 53]. IC_50_ values against the various HDAC activities were determined using nonlinear regression in Prism 8.0 software (GraphPad Software, Inc., La Jolla, CA, USA).

### Docking studies

Docking studies were carried out as previously reported [54] using the Protein Data Bank (PDB) codes 4BKX, 4LY1, 4A69, 2VQM, 5EDU, 3C10, and 3EW8 corresponding to HDAC1, HDAC2, HDAC3, HDAC4, HDAC6, HDAC7, and HDAC8, respectively. MAKV-6, MAKV-7, MAKV-8, MAKV-10, and MAKV-12, and SAHA were drawn using ChemDraw Professional software version 15.0 (PerkinElmer Informatics).

### Prediction of in silico drug-likeliness parameters

The web-based Molinspiration Cheminformatics (http://www.molinspiration.com/products.html) and PreADMET ver2.0 (https://preadmet.bmdrc.kr) programs were used to compute compound drug-likeness parameters.

### Cell viability and proliferation test

Cell viability and proliferation were evaluated using the Trypan Blue exclusion method (BioWhittaker ®). Cells were processed using a semi-automated image-based cell analyzer (Cedex XS Innovatis, Roche, Luxembourg, Luxembourg), which provided the cell number as well as cell viability based on the fraction of trypan blue-positive cells.

For colony formation assays, 1000 cells were grown in 1 mL of semi-solid methylcellulose medium (Methocult H4230, StemCell Technologies Inc., Grenoble, France) supplemented with 10% heat-inactivated FCS (BioWhittaker ®) in 12-well plates. Colonies were detected after 10 days of culturing by adding 5 mg/mL 3-(4,5-dimethylthiazol-2-yl)-2,5-diphenyltetrazoliumbromide (MTT) reagent (Sigma-Aldrich) and incubating for 15 min at 37 °C. Pictures were taken with the GelDoc XR+ System (BioRad, Temse, Belgium), and quantifications were conducted using Image J software (US National Institute of Health, Bethesda, MD, USA).

### Protein extractions and western blotting analysis

Cells were lyzed in 10% (v/v) Mammalian Protein Extraction Reagent solution (MPER®; Thermofisher, Erembodegen, Belgium) supplemented with 1× protease inhibitor cocktail (Complete EDTA-free, Roche) according to the manufacturer’s instructions. Histone enrichment was performed in acidic conditions as previously described [55]. The protein concentration was determined using the Bradford assay (BioRad).

Western blotting was performed as previously described [53] using the following primary antibodies: acetylated α-tubulin (sc-23950, RRID: AB_628409, 1/1000), ATF6 (sc-166659, RRID: AB_2058901, 1/250), c-ABL (sc-23, RRID: AB_626775, 1/1000), caspase 3 (sc-56053, RRID: AB_781826, 1/1000), GRP78 (sc-13968, RRID: AB_2119991, 1/1000), p62/SQSTM1 (sc-28359, RRID: AB_628279, 1/1000), and phosphorylated PERK (sc-32577, RRID: AB_2293243, 1/1000) from Santa Cruz Biotechnology (Boechout, Belgium); phosphorylated H_2_AX (05-636, RRID: AB_2755003, 1/500), acetylated histone H4 (06-866, RRID: AB_310270, 1/50000), and histone H1 (05-457, RRID: AB_310843, 1/2000) from Millipore; α-tubulin (CP06, RRID: AB_2617116, 1/5000) from Calbiochem; β-actin (A5441, RRID: AB_476744, 1/20000) and LC3 (L7543, RRID: AB_796155, 1/1000) from Sigma-Aldrich; beclin-1 (3738, RRID: AB_490837, 1/1000), caspase 7 (9494, RRID: AB_2068141, 1/1000), caspase 8 (9746, RRID: AB_2275120, 1/1000), caspase 9 (9502, RRID: AB_2068621, 1/1000), eIF2α (9722, RRID: AB_2230924, 1/2000), MCL-1 (4572, RRID: AB_2281980, 1/1000), PARP-1 (9542, RRID: AB_2160739, 1/1000), PERK (3192, RRID: AB_2095847, 1/1000), phosphorylated BCR (3901, RRID: AB_2063779, 1/1000), phosphorylated eIF2α (3898, RRID not available, 1/2000), STAT5 (9363, RRID: AB_2196923, 1/5000), and phosphorylated STAT5 (9351, RRID: AB_2315225, 1/1000) from Cell Signaling (Leiden, Netherlands); and c-Myc (51-1485GR, RRID: AB_2148606, 1/250) from BD Biosciences (San Jose, CA, USA). Corresponding secondary antibodies were obtained from Santa Cruz Biotechnology. Western blot quantifications were performed with the ImageQuant TL software (GE Healthcare), and corresponding fold change values reported to control are indicated underneath western blot pictures, unless otherwise specified. The EC_50_ values, which represent 50% of the maximum effect, were calculated using Prism 8.0 software.

### Cell cycle analyses

Cells were fixed at 4 °C for 1 h with 70% ethanol and stained for 20 min with 1 μg/mL propidium iodide in 1× PBS supplemented with 100 μg/mL RNase A (Roche). Stained samples were processed through a cytometer (FACS Calibur, BD Biosciences), and data were recorded statistically (10,000 events/sample) using the CellQuest Pro software (BD, Biosciences). The percentage of cells in each phase of the cycle was finally determined by the Dean-Jet-Fox algorithm using the Flow-Jo 8.8.5 software (Tree Star, Inc., Ashland, OR, USA).

### Cell death assessment

Nuclear morphology was evaluated under an IX81 (MT10) fluorescent microscope (Olympus, Aartselaar, Belgium) using the Cell^M software on cells incubated with 1 μg/mL Hoechst 33342 (Invitrogen, Tournai, Belgium) for 15 min at 37 °C and 1 μg/mL propidium iodide (Sigma-Aldrich).

Phosphatidylserine exposure was evaluated using the FITC Annexin V Apoptosis Detection Kit I (BD Biosciences) according to the supplier’s instructions. Stained samples were processed through a cytometer (FACS Calibur, BD Biosciences), and data were recorded statistically (10,000 events/sample) using the CellQuest Pro software (BD, Biosciences). Data were analyzed using the Flow-Jo 8.8.5 software.

### Determination of mitochondrial membrane potential

The MMP was assessed by staining with MitoTracker® Red CMXRos (Invitrogen) according to the manufacturer’s protocol. Stained samples (20,000 events/sample) were processed through a cytometer (FACS Calibur, BD Biosciences).

### Assessment of gene expression

The total RNA was extracted with the NucleoSpin® RNA Plus Kit (Macherey-Nagel, Hoerdt, France) according to manufacturer’s instructions. Reverse transcription and real-time PCR were performed as previously described [56]. The following primers (Eurogentec, Liège, Belgium) were used: β-actin (forward 5′-CTCTTCCAGCCTTCCTTCCT-3′, reverse 5′-AGCACTGTGTTGGCGTACAG-3′); DDIT3 (forward 5′-TGGAAGCCTGGTATGAGGAC-3′, reverse 5′-AAGCAGGGTCAAGAGTGGTG-3′).

XBP1 splicing analysis was performed by end-point PCR as described previously [57] using the following primers (Eurogentec; forward: 5′- GGAGTTAAGACAGCGCTTGG -3′, reverse: 5′- ACTGGGTCCAAGTTGTCCAG -3′).

### Morphological analysis

After staining cells with the Diff-Quick Stain Kit (Dade Behring S.A., Brussels, Belgium), morphological analyses were performed as previously described [56].

### Visualization and quantification of autophagic vesicles

For fluorescence microscopy, the Cyto-ID® Autophagy Detection Kit (Enzo Life Science) was used according to the manufacturer’s instructions. Stained cells from three biological replicates were visualized under an IX81 (MT10) fluorescent microscope. Green fluorescent autophagy-related organelles (pre-autophagosomes, autophagosomes, and autophagolysosomes) were quantified as green signal area per cell nucleus as advised by the manufacturer. Segmentation of these areas was based on 31 parameters assessing color, texture, and edge and was carried out in Ilastik, version 1.3.0 developed by the European Molecular Biology Laboratory, Heidelberg [58]. Classifiers trained for these parameters on a set of representative images were then applied to batch process multiple images as described in Ilastik’s user manual. Binary masks thus obtained were measured in FIJI [59] after applying a size filter to remove small size artifacts resulting from segmentation.

Transmission electron microscopy was performed as previously described [60].

### Evaluation of cellular ALDH activity

Cellular ALDH activity was assessed using the ALDEFLUOR^TM^ kit (StemCell Technologies Inc.) according to the manufacturer’s procedure. Diethylaminobenzaldehyde, a specific inhibitor of ALDH activity, was used to differentiate cells with low or high ALDH activity. Stained samples (100,000 events/sample) were processed through a cytometer (FACS Calibur, BD Biosciences).

### Transfections

Transfections were carried out with 1.5 μL HiPerFect Transfection reagent (Qiagen, Venlo, Netherlands) and 1 nM siRNAs (Qiagen) either targeting the human beclin-1 gene [Hs_BECN1_2 (SI00055580)] or non-targeting (AllStars Negative Control) as described elsewhere [61]. Treatment compounds were added to the culture medium 24 h post-transfection.

### Zebrafish cancer cell xenografts

Cancer xenograft assays in zebrafish were performed as previously described [62] using cells stained with 2 μM CM-Dil (Invitrogen) and diluted in 1× PBS supplemented with 1% phenol red sodium salt solution.

### Statistical analyses

All histograms represent the mean ± standard deviation (SD) of at least 3 independent experiments. Significant differences were determined using one-way or two-way analyses of variance (ANOVA) followed by the Holm-Sidak’s multiple comparison tests in the Prism 8.0 software. Variations between patient and Cyto-ID-stained samples were evaluated using two-tailed Welch *t* test in the R 3.6.0 software and one-tailed Mann-Whitney *U* test in the Prism 8.0 software, respectively. Statistical significances were evaluated with *p* values below 0.05 and are represented by the following legend: *p ≤ 0.05, **p ≤ 0.01, ***p ≤ 0.001. In all experiments, data were presented as the mean ± standard deviation, and results of treated samples were statistically compared to the corresponding vehicle unless otherwise specified.

The CI was calculated according to Chou and Talalay [63] using the Compusyn Software (ComboSyn, Inc., Paramus, NJ, USA). CI values below or above 1 indicate synergism or antagonism, respectively, whereas the effect is determined to be additive when the CI is equal to 1.

## Supplementary information

**Additional file 1: Figure S1.** Reaction scheme showing the synthesis of MAKV-6, -7, -8, -10 and -12. **Figure S2.** Docking of MAKV-8 into human HDAC isoenzymes. **Figure S3.** Effect of MAKV-8 derived compounds on *in vitro* HDAC6 and total HDAC activities. **Figure S4.** Effect of imatinib treatment on KBM-5R cell death. **Figure S5.** Effect of pan-HDACi MAKV-8 and SAHA on healthy model viability. **Figure S6.** Effect of imatinib treatment on replicative ability of imatinib-sensitive and -resistant CML cells. **Figure S7.** Panel of zebrafish pictures.

## Data Availability

The datasets generated and/or analyzed during the current study are available in the Mendeley repository, https://data.mendeley.com/datasets/x25w3bpcyk/draft?a=41167977-f905-442b-9c79-c7dfab18295f.

## Abbreviations

ABL: Abelson murine leukemia viral oncogene homolog 1
ALDH: Aldehyde dehydrogenase
ATF: Activating transcription factor
BCR: Breakpoint cluster region
CI: Combination index
CML: Chronic myeloid leukemia
DDIT: DNA damage-inducible transcript
DMSO: Dimethylsulfoxide
ER: Endoplasmic reticulum
eIF: Eukaryotic initiation factor
FCS: Fetal calf serum
GRP78: 78 kDa glucose-regulated protein
γH2AX: H2AX phosphorylation
HDAC: Histone deacetylase
HDACi: HDAC inhibitor
HSC: Healthy stem cell
LC: Microtubule-associated protein 1 light chain
LSC: Leukemic stem cell
MCL: Myeloid cell leukemia
MMP: Mitochondrial membrane potential
PARP: Poly (ADP-ribose) polymerase
PBMC: Peripheral blood mononuclear cell
PDB: Protein Data Bank
PERK: Protein kinase RNA-like ER kinase
SAHA: Suberoylanilide hydroxamic acid
siRNA: Small interfering RNA
SQSTM: Sequestosome
STAT: Signal transducer and activator of transcription
TKi: Tyrosine kinase inhibitor
UPR: Unfolded protein response
XBP: X-box binding protein
