# Supplementary material for: Novel HDAC inhibitor MAKV-8 and imatinib synergistically kill chronic myeloid leukemia cells via inhibition of BCR-ABL/MYC-signaling: effect on imatinib resistance and stem cells
Source: Clin Epigenetics. 2020 May 19;12:69. doi: 10.1186/s13148-020-00839-z (PMC7236970; doi:10.1186/s13148-020-00839-z)
Supplement: Supplementary file 1 — Additional file 1: Figure S1. Reaction scheme showing the synthesis of MAKV-6, -7, -8, -10 and -12. Figure S2. Docking of MAKV-8 into human HDAC isoenzymes. Figure S3. Effect of MAKV-8 derived compounds on in vitro HDAC6 and total HDAC activities. Figure S4. Effect of imatinib treatment on KBM-5R cell death. Figure S5. Effect of pan-HDACi MAKV-8 and SAHA on healthy model viability. Figure S6. Effect of imatinib treatment on replicative ability of imatinib-sensitive and -resistant CML cells. Figure S7. Panel of zebrafish pictures. [file 13148_2020_839_MOESM1_ESM.docx]

**Supplementary information**

**Novel HDAC inhibitor MAKV-8 and imatinib synergistically kill chronic myeloid leukemia cells via inhibition of BCR-ABL/MYC-signaling: effect on imatinib resistance and stem cells**

Manon Lernoux^1^, Michael Schnekenburger^1^, Hélène Losson^1^, Koen Vermeulen^2^, Hyunggu Hahn^3^, Déborah Gérard^1^, Jin-Young Lee^3^, Aloran Mazumder^3^, Muneer Ahamed^2^, Christo Christov^4^, Dong-Wook Kim^5^, Mario Dicato^1^, Guy Bormans^2^, Byung-Woo Han^3*^, Marc Diederich^3*^

Figure S1: Docking of MAKV-8 into human HDAC isoenzymes.

Docking poses of MAKV-8 molecule, represented as stick model (orange), on the crystal structure of the indicated HDAC isoenzymes that are superposed (white; PDB codes: 4BKX, 4LY1, 4A69, 2VQM, 5EDU, 3C10, and 3EW8 for HDAC1, HDAC2, HDAC3, HDAC4, HDAC6, HDAC7 and HDAC8, respectively). Nitrogen and oxygen are colored in blue and red, respectively.

Figure S2: Effect of MAKV-8 derived compounds on *in vitro* HDAC6 and total HDAC activities.

*In vitro* HDAC activity assays were conducted with increasing concentrations of MAKV-8 derived compounds. Relative activities of total HDAC and HDAC6 were determined by comparison to the vehicle DMSO and correspond to the mean ± SD of three independent experiments for MAKV-6 and MAKV-7, or two independent experiments for MAKV-10 and MAKV-12.

Figure S3: Effect of imatinib treatment on KBM-5R cell death.

KBM-5R cells were treated with increasing concentrations of imatinib and nuclear morphology was studied after 48 hours of treatment.

Figure S4: Effect of pan-HDACi MAKV-8 and SAHA on healthy model viability.

PBMCs (upper panel) and platelets (lower panel) were treated with the indicated concentrations of MAKV-8 or SAHA for 48 hours. Cell viability was assessed based on the Trypan Blue exclusion method for PBMCs and by flow cytometry after Annexin V staining for platelets.

Figure S5: Effect of imatinib treatment on replicative ability of imatinib-sensitive and -resistant CML cells.

Imatinib-sensitive and -resistant CML cells were grown in semisolid methylcellulose medium in presence of increasing concentrations of imatinib for 10 days and colony formation was then scored after addition of MTT. Quantifications of total MTT intensity correspond the mean ± SD of three independent experiments.


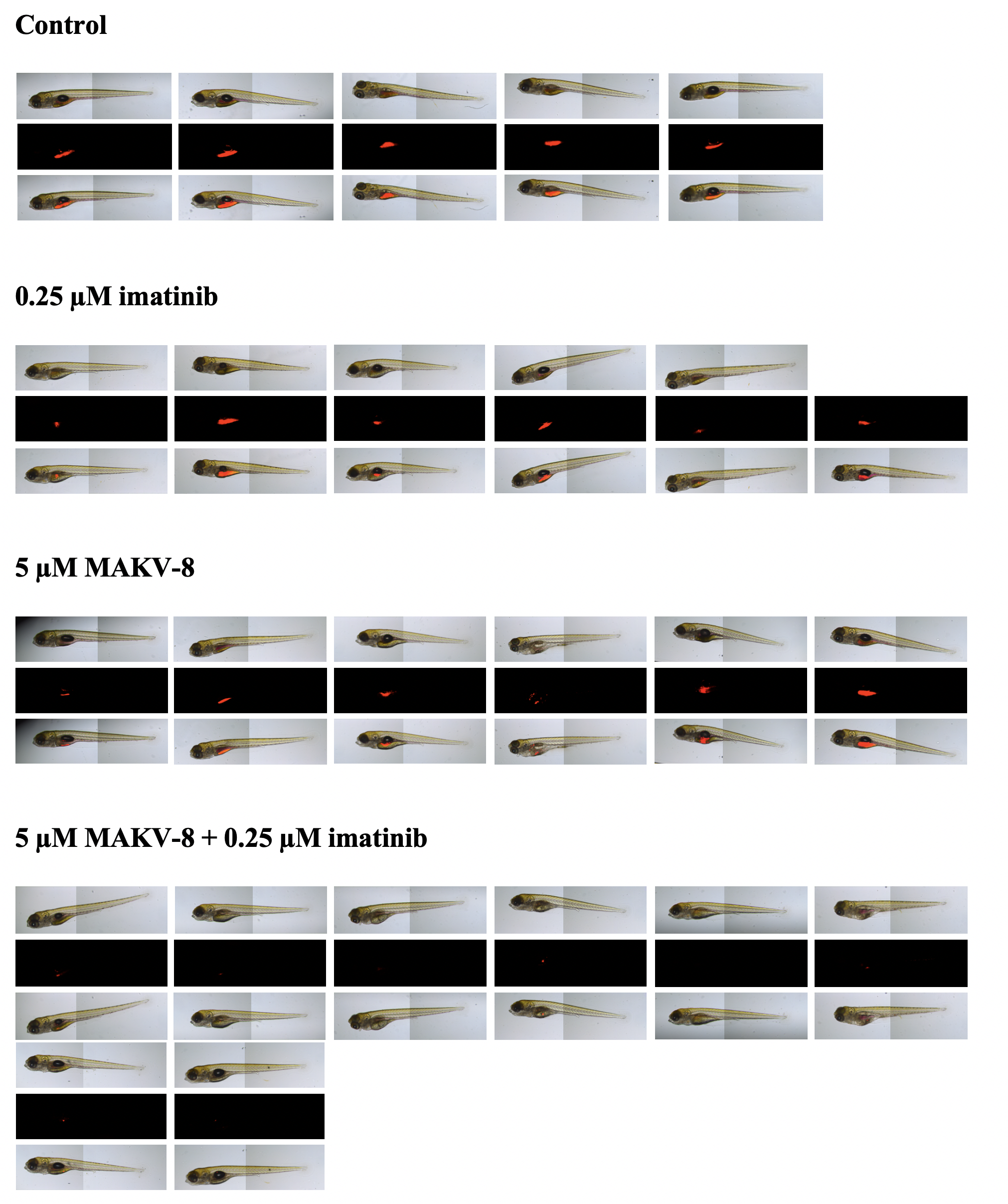


Figure S6: Panel of zebrafish pictures.

K-562 cells were treated as indicated in the figure for 24 hours, labeled with fluorescent dye and then injected in the zebrafish yolk sac. All zebrafish pictures for each condition are shown. Upper, middle and lower pictures represent bright field, red fluorescence and merge, respectively.

Figure S7: Reaction scheme showing the synthesis of MAKV-6, -7, -8, -10 and -12.
